# Aged (Black) versus Raw Garlic against Ischemia/Reperfusion-Induced Cardiac Complications

**DOI:** 10.3390/ijms19041017

**Published:** 2018-03-28

**Authors:** Attila Czompa, Kitti Szoke, Jozsef Prokisch, Alexandra Gyongyosi, Istvan Bak, Gyorgy Balla, Arpad Tosaki, Istvan Lekli

**Affiliations:** 1 Department of Pharmacology, Faculty of Pharmacy, University of Debrecen, 4012 Debrecen, Hungary; czompa.attila@pharm.unideb.hu (A.C.); szoke.kitti@pharm.unideb.hu (K.S.); gyongyosi.alexandra@pharm.unideb.hu (A.G.); bak.istvan@pharm.unideb.hu (I.B.); tosaki.arpad@pharm.unideb.hu (A.T.); 2 Department of Animal Husbandry, Institute of Animal Science, Biotechnology and Nature Conservation, Faculty of Agriculture, Food Science and Environmental Management, University of Debrecen, 4012 Debrecen, Hungary; jprokisch@agr.unideb.hu; 3 Department of Pediatrics, Medical and Health Science Center, University of Debrecen, 4012 Debrecen, Hungary; balla@med.unideb.hu; 4 Hemostasis, Thrombosis and Vascular Biology Research Group, Hungarian Academy of Sciences, 4012 Debrecen, Hungary

**Keywords:** garlic, ischemia, heme oxygenase, reperfusion, heart

## Abstract

Recent evidence from studies suggests that aged black garlic also has an effect on health. The major aim of the present study is to compare the effect of raw and aged black garlic on postischemic cardiac recovery. Male Sprague Dawley rats were randomly divided into three groups. Animals of the first group were fed with raw garlic, animals of the second group received aged black garlic, while the third group served as vehicle-treated controls. Upon conclusion of the treatment, isolated hearts were undertaken to ischemia/reperfusion. Heart function and infarct size were measured and the level of HO-1 and iNOS were studied. Superior postischemic cardiac function and reduced infarct size in both garlic treated groups compared to the drug-free control group, indicated cardioprotective effects. However, no significant differences between the garlic treated groups were observed. Western blot analysis revealed that raw garlic enhanced the level of HO-1 before ischemia, while in ischemic samples, we found elevated HO-1 expression in both garlic treated groups. The level of iNOS was the same before ischemia in all groups, however, a markedly reduced iNOS level in ischemic/reperfused hearts originating from control and raw garlic treated animals was observed. Samples from aged black garlic treated animals demonstrated that the level of iNOS was not significantly reduced after ischemia/reperfusion. Taken together these results indicate that not only raw but also aged black garlic possess a cardioprotective effect.

## 1. Introduction

Raw garlic (RG) has a long history as a spice and as a medical plant. It is mentioned in ancient literature from different parts of the Word including Egypt, India and Greece. Moreover, different cultures without any connection were associated with similar beneficial effects on for garlic, as summarized by Rivlin [1]. An example is ancient athletes during Olympic Games used garlic to enhance their endurance. Furthermore, garlic bulbs were found in the pyramid of Tutankhamen [2]. Many health-related effects were later confirmed by modern evidence-based medicine. Recent evidence suggests that a processed form of garlic called “Aged black garlic” (ABG) also possesses beneficial to health properties including antidiabetic and antiatherogenic effects [3]. 

According to the Framingham Heart Study, both diabetes and perturbed lipid homeostasis might contribute to the pathogenesis of ischemic heart diseases [4,5]. Indeed, consequences of atherosclerosis and micro- and macrovascular complications of diabetes often lead to an impaired medical state. Different studies have shown that the consumption of medicinal plants can help to normalize glucose and fat homeostasis. Through normalization of metabolic homeostasis, these plants can indirectly protect the heart from ischemia, for example decreasing the blood sugar level or normalizing the lipid homeostasis, which can reduce the risk of ischemic heart diseases [6], or directly possessing a cardioprotective effect acting on cardiomyocytes or endothelial cells in cardiac vessels. The direct cardioprotective effect might relate to the antioxidant properties of plants, or a direct effect on ion channels or induction of different stress related proteins such as hemoxygenase-1 (HO-1). Indeed, lower levels of HO-1 mRNA and endogen CO production are found to be related to increased risks of reperfusion induced ventricular fibrillation [7]. Furthermore, upregulation of HO-1 by plants or their extracts play a critical role in the prevention of I/R-injury in different organs [8,9]. Recently, ABG was shown to induce HO-1 in an epithelial cell model, which might indicate a tissue protective property of ABG [10]. The major objective of the present study was to compare the effect of ABG and raw garlic (RG) on the pre- and postischemic cardiac function and investigate the underlying molecular mechanisms. 

## 2. Results

### 2.1. Analysis of the Composition of Raw and Aged Black Garlic

**Figure 1:**
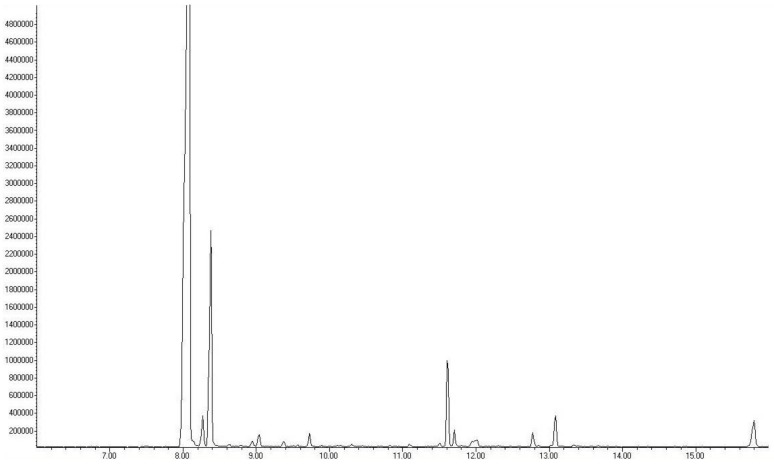

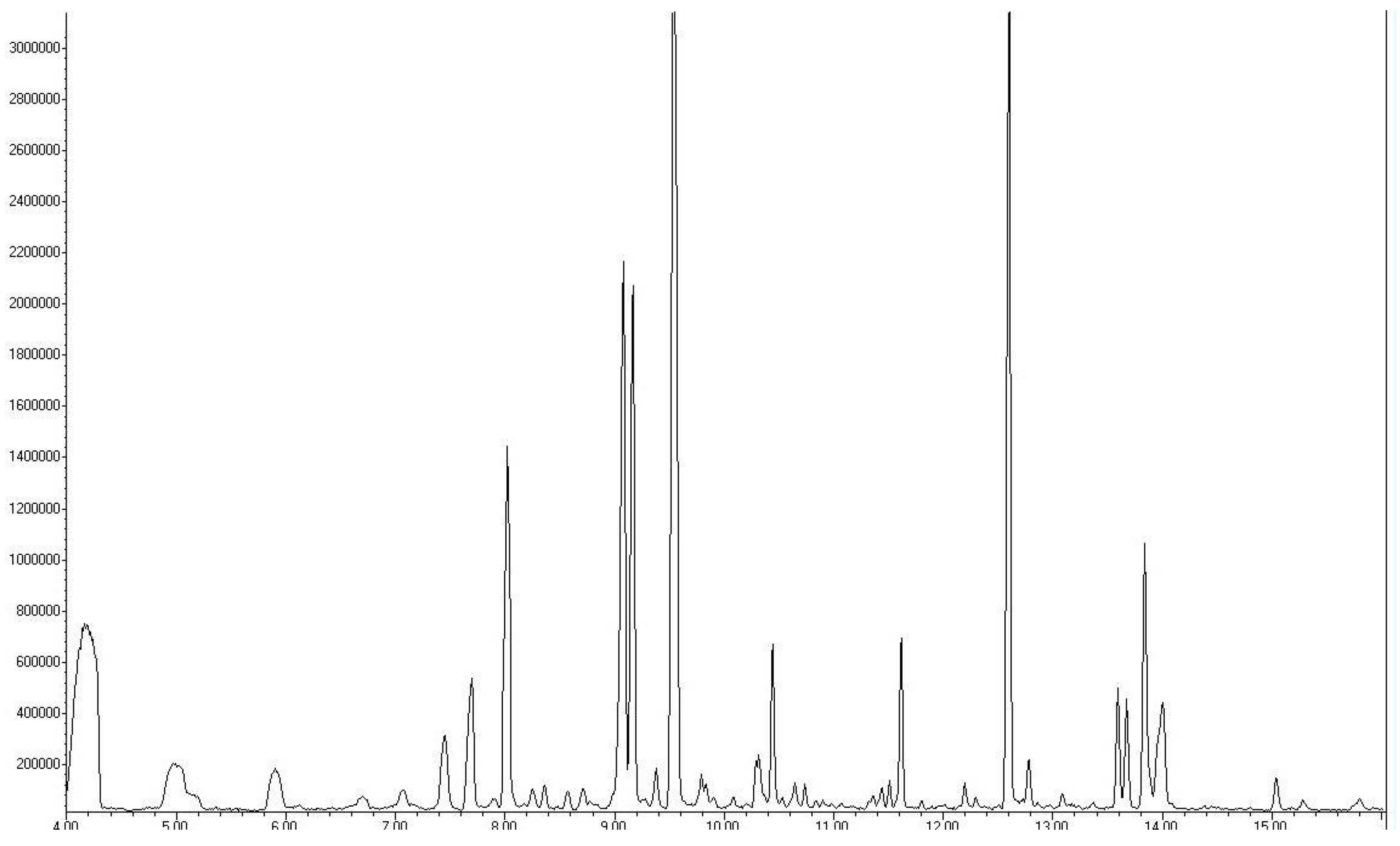
Representative chromatograms of raw garlic (**upper panel**) and black garlic (**lower panel**) Headspace-SPME technique was used to obtained samples followed by GC-MS analysis.

Figure 1 and Table 1 depict the different sulfur-containing molecules in both garlic preparations. It must be noted that amounts and numbers of different sulfur-containing compounds are higher in ABG. The study failed to detect any allicin in ABG (Table 1). During the aging process, the Maillard reaction occurred, which was evidenced by the presence of the 2-acetyl-1-pyrroline in ABG and its absence in raw garlic.

### 2.2. Effect of Raw and Aged Garlic on Body Weight and Blood Enzymes

**Figure 2:**
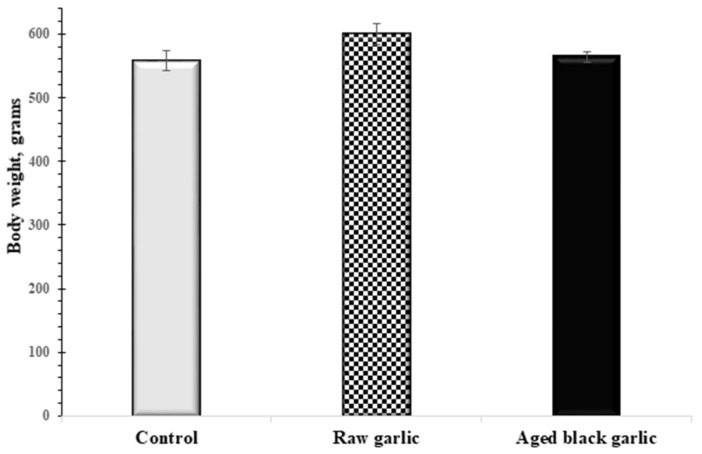
The effects of raw and aged black garlic on bodyweight. Upon conclusion of the treatment with raw (RG) and aged black garlic (ABG) bodyweight was measured. Results are expressed as mean ± SEM. *n* = 9 in each group.

No significant differences in body weight were seen after 4 weeks of RG- and ABG-treated groups compared to the vehicle-treated group (Figure 2). Following the treatment period with different garlic preparations, peripheral blood was collected, and various blood enzymes were measured. Table 2 shows that the levels of the investigated blood markers remained in the normal range in all groups. 

### 2.3. Raw and Aged Garlic Treatment Protect the Heart from Ischemia/Reperfusion Injury 

**Figure 3:**
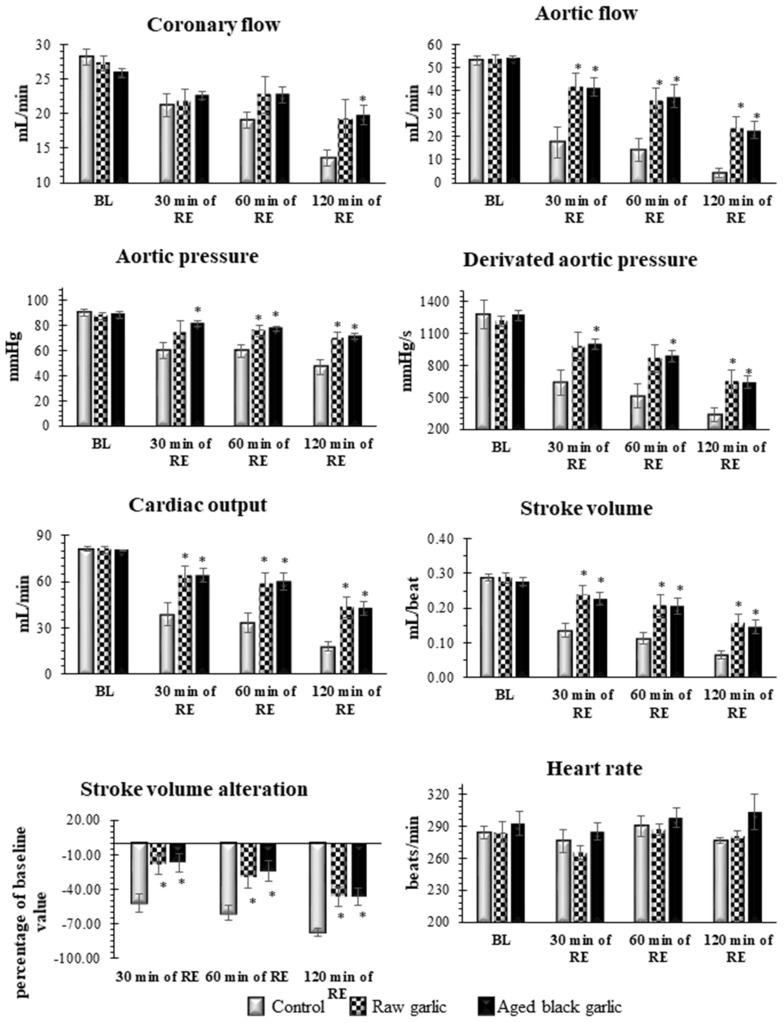
Effects of raw and aged black garlic on pre and postischemic cardiac functions. Following the end of the treatments, isolated hearts were subjected to 30 min of ischemia and 120 min of reperfusion. Pre- and postischemic left ventricular functions including heart rate (HR), coronary flow (CF), aortic flow (AF), aortic pressure (AOP), the first derivative of aortic pressure (AOdP/dt), stroke volume (SV), alteration in stroke volume were studied. Results are expressed as mean ± SEM *n* = 9 in each group. Raw garlic (RG); aged black garlic (ABG). * *p* < 0.05 in comparison with the control parameters within the same period.

To compare the beneficial to health effects of RG and ABG garlic, isolated hearts originating from treated animals were taken to 30 min of global ischemia and 120 min of reperfusion. No significant differences were seen in preischemic values of the studied cardiac functions including CF, AF, AOP, AOdP/dt, CO, and SV alteration. However, Figure 3 shows significantly increased postischemic cardiac function in the presence of raw and aged garlic, respectively, was detected. Thus, after 30 min of ischemia and 120 min of reperfusion, AF was significantly enhanced in the treated groups compared to their control value of 4.3 ± 2.0 mL to 24.1 ± 4.6 mL for raw garlic, and 22.7 ± 3.8 mL for aged garlic. Interestingly, in comparison with the control values of 13.6 ± 1.2 mL superior CF values in the treated groups was observed, in the raw garlic group a value of 19.3 ± 2.8 mL and for aged garlic a value of 19.9 ± 1.4 mL, respectively. 

### 2.4. Infarct Size Reduction by Raw and Aged Black Garlic Treatment in Ischemic Reperfused Myocardium

**Figure 4:**
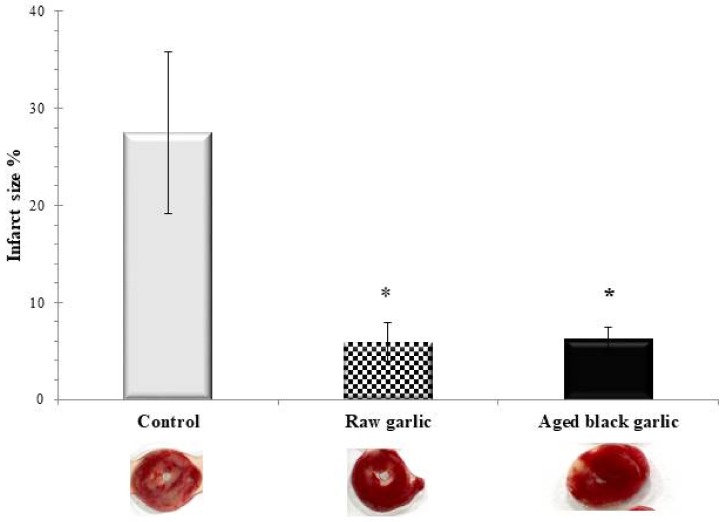
Effects of raw and aged black garlic on infarct size. Following ischemia/reperfusion, hearts were perfused with TTC solution via aortic cannula to assess the infarcted size (infarcted area in white, and non-ischemic region stained in red. Results are expressed as mean ± SEM. *n* = 4 in each group. Raw garlic (RG); Aged black garlic (ABG). * *p* < 0.05 in comparison with the control group.

To further confirm the cardioprotective effect of the RG and ABG at the end of I/R TTC, staining was carried out to monitor the size of infarcted tissue. It is depicted in Figure 4 that the 27.5 ± 8.4% infarcted volume was in the control group, and both raw and aged garlic were able to significantly reduce the infarct volume to 5.9 ± 2.0% and 6.2 ± 1.2%, respectively. There were no statistically significant differences between the two garlic treated groups. 

### 2.5. Induction of HO-1 and iNOS by Garlic 

**Figure 5:**
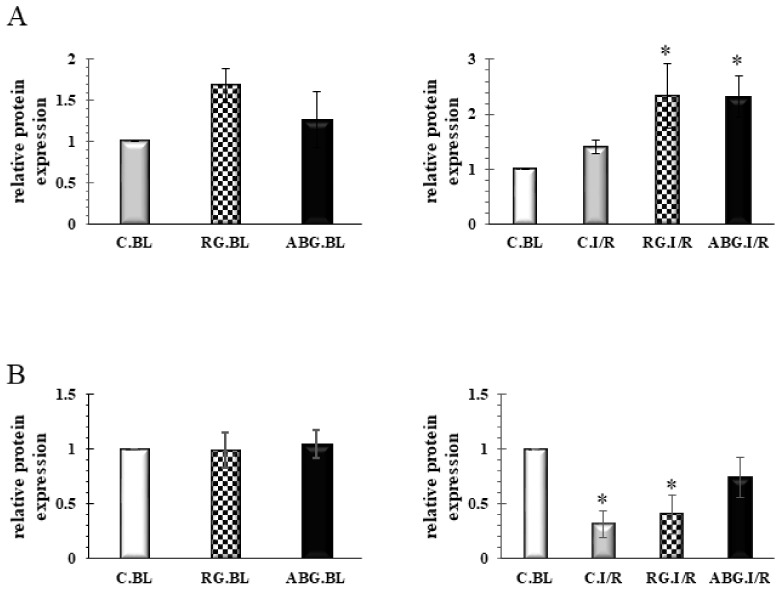
Effect of raw and aged black garlic on HO-1 and iNOS expression. Panel (**A**): Protein samples from hearts originating from animals treated with RG, ABG or vehicle were separated, blotted and probed with the HO-1 antibody. Panel (**B**): Protein samples from hearts originating from animals treated with RG, ABG or vehicle were separated, blotted and probed with iNOS antibody. Results are expressed as mean ± SEM *n* = 5–7. Raw garlic (RG); aged black garlic (ABG). BL: baseline samples; I/R: ischemia/reperfused samples. * *p* < 0.05 in comparison with the control baseline parameters (CBL).

To explore the molecular mechanisms by which RG and ABG protected the heart against I/R injury, the levels of HO-1 and iNOS were measured by Western blot. Figure 5A illustrates that the study detected the enhanced level of HO-1 for RG preparation before I/R. However, after I/R, in both garlic treated groups, a significantly elevated level of HO-1 was observed. The evaluation of iNOS is depicted in Figure 5B and no significant differences were seen before I/R between the groups. However, after I/R a significantly reduced protein level of iNOS was seen in the control and RG treated hearts, but AGB was able to prevent the reduction of iNOS after I/R. Representative blots are provided as supplementary Figure S1.

## 3. Discussion

A healthy diet is being considered as a keystone of different stages of primary, secondary or tertiary prevention. Nowadays, the so-called functional food is gaining importance in different stages of prevention or might be used as complementary treatment next to medicines. Garlic is being considered as a very healthy spice or medical plant, however, consumption of RG is limited by the intense taste, the foul breath, and the body odor. Even consumption of garlic extracts might have the same effect on body odor. The taste and effect on breath by ABG are very moderate compared to RG. Furthermore, there are preparations of ABG with honey or chocolate, which are very tasty. Intact garlic is abundant in γ-glutamyl cysteine, which can be transformed to alliin. During culinary preparations like cutting, crushing etcetera, alliin is converted to the odiferous allicin by alliinase [11]. During the aging process, when garlic is exposed to a relatively high temperature (70 °C) and high humidity, the major sulfur-containing compound γ-glutamyl cysteine is converted to *S*-allylmercaptocysteine (SAC), which is a major water-soluble antioxidant compound of ABG [12]. These results also verify that during aging, the alliin is being converted to other sulfur-containing compounds and even the level of allicin in ABG remains under the limit of detection. Furthermore, under such conditions, the Maillard reaction also occurs when sugars react with amino acids leading to brownish colors. The compound 2-acetyl-1-pyrroline is present in some plants in raw form; however, it has been shown that during cooking, it forms as a product of the Maillard reaction [13,14]. This study failed to find 2-acetyl-1-pyrroline in RG, however, we were able to detect it in ABG samples, indicating that under this study’s conditions, the Maillard reaction also contributes to the “aging and transformation” of RG to ABG. Figure 1 shows that the authors identified different sulphur-containing molecules in both garlic preparations, which might play a role in cardioprotection and possess beneficial to health effects as suggested earlier [15,16]. However, based on these results the authors cannot pick up only one single compound. 

The major aim of the present study was to compare the cardiovascular effect of raw and aged black garlic in an experimental model. Consistent with earlier studies [17,18,19], these results clearly demonstrate that both RG and ABG possess similar very significant cardioprotective effects in I/R-ed myocardium, as evidenced by superior postischemic cardiac functions and smaller infarct size. The study failed to find any significant differences between the two treated groups, indicating that the aging process of garlic does not alter the cardioprotective ability of the preparation. Indeed, different studies have compared the antioxidant and anti-inflammatory effects of RG and ABG under certain conditions [20,21]. Lee and colleagues have shown superior antioxidant properties for ABG over RG in diabetic animals [21]. Earlier, a long term garlic administration was shown to enhance the level of endogen antioxidants such as catalase, SOD in the myocardium in a dose dependent manner [19]. The authors have suggested the contribution of the enhanced antioxidant defense mechanism to the cardioprotective ability of garlic. Consistently, enhanced antioxidant activity was found in ABG by Jeong and co-workers, however, the antioxidant capability was not directly proportional to the anti-inflammatory property of the different garlic preparations in an LPS-stimulated inflammatory model [20]. The authors found that pyruvate and other polyphenols, flavonoids and organosulfur compounds, enriched during the aging process, act synergistically as antioxidants in ABG. Furthermore, the anti-inflammatory effects of pyruvate found in ABG might be perturbed by the sugar component in ABG.

The study did not find any analyzed biomarkers out of the physiological range; since all remained under the physiological level any effect on metabolism cannot be concluded. However, it must be noted that the possibility is quite reasonable since rats were also symptom-free during the treatment period. The same reasoning might explain the unaltered body weight during the experiments. Thus, the metabolic effect of the garlic preparations should be studied in a diabetic or atherosclerotic animal model. 

To explore the molecular mechanisms by which the different garlic preparations protect the heart, the levels of HO-1 and iNOS were studied. The study found that treatment with RG can enhance the level of HO-1 before ischemia. Following ischemia in both garlic-treated groups, the level of HO-1 is significantly enhanced. Induction of HO-1 leads to the production of Fe^2+^, endogen CO and biliverdin/bilirubin as a byproduct of heme metabolism. Enhanced activity of the HO-1/CO system by different natural products has been shown to induce cardioprotection [8,22]. Upregulation of HO-1 might be an adaptive response to different harmful stimuli such as ischemia, or even excess heme levels. Recently, it has been suggested that under special conditions, an excess level of HO-1 and its by-products might fail to protect against I/R. Not long ago, the authors reported that cardioprotection induced by a low dose beta-carotene treatment is absent at a high dose beta-carotene treatment; although in both cases, an enhanced level of HO-1 was observed. The authors have speculated that, probably, the high level of Fe^2+^ in the presence of a high amount of beta-carotene might behave as a pro-oxidant [23]. However, in that circumstance, the authors assumed that mild HO-1 induction was a contributing factor to the cardioprotective effect of HO-1. Earlier, sour cherry seed extract was found to protect the heart via upregulation of HO-1 protein [8]. Ginseng derived ginsenoside was proven to activate the Nrf2/HO-1 pathway and protect H9c2 cells against hypoxia/reoxygenation in a recent study [24]. Furthermore, Issan and colleagues have demonstrated that pharmacological induction of HO-1 by CoPP protects H9c2 cells against hypoxia and also diabetic hearts from ischemia via the modulation of the AKT/GSK3β pathway [25]. However, it must be noted that from this study’s results, the authors cannot conclude which component is the major HO-1-inducer since the composition of the two garlic preparations is not the same.

NOSs, possessing three isoforms including eNOS, nNOS, and iNOS, are a group of enzymes producing NO, which is a gaseous transmitter playing a role in different physiological and pathophysiological processes. During the authors’ experiments, the level of iNOS was significantly reduced after I/R in the vehicle-treated control and RG-treated groups. A slight decrement was observed in the ABG group, however, it was not at a significant level indicating the ability of ABG to prevent iNOS loss after I/R. Recently, an extract of ABG was shown to possess dose-dependent cardioprotective effects and similarly, an enhanced iNOS expression was found in ABG-treated hearts [15]. Similarly, in a recent study, a reduced level of iNOS mRNA expression was found in hearts obtained from saline-treated animals after infarction. However, fish oil treatment prevented the decreased expression of iNOS after infarction [26]. Furthermore, enhanced expression of iNOS accompanied by smaller infarct size were found in eNOS KO animals in response to I/R-injury in an “ex vivo” model, indicating that NO plays a role in cardioprotection [27]. It must be noted that the authors did not observe any alteration of iNOS expression in WT animals after I/R. Quite the reverse, as “in vivo” studies showed earlier, overexpression of iNOS might contribute to myocardial injury [28,29]. Thus, cardioprotection afforded by Propofol and Sabiporide (Na^+^/H^+^ exchanger-1 inhibitor) was found to be mediated via the suppression of iNOS. Furthermore, slightly enhanced expression of iNOS was detected in hearts obtained from overfed animals [30]. Moreover, enhanced iNOS activity was found to play a role in cardiomyocyte dysfunction in a regional ischemia in vivo [31]. Interestingly, in another study, enhanced expression of iNOS was observed in the ischemic region of the heart after a “sub-lethal ischemic” insult suggesting a regulatory role of iNOS during the late preconditioning [32]. Based on the above-mentioned outcomes, the role of NO and NOSs in ischemic tissue could be both beneficial and harmful, depending on the environment or tissue damage [33,34]. However, it must be noted that the difference in iNOS expression also might arise from the different experimental models since, in the current study, “ex vivo” 30 min ischemia and 120 min of reperfusion was used to mimic I/R, while in other studies “in vivo” 6–24 h of reperfusion were allowed. Under “in vivo” conditions, the authors cannot rule out the influence of the immune system and platelets on the expression of different proteins [27]. 

Taken together, this study’s results clearly demonstrate that the components of garlic during the aging process are altered, but ABG still possesses beneficial health effects. The outcome of the present study demonstrates that consumption of ABG is also healthy and could reduce I/R-induced cardiac complications. However, further studies need to be carried out to study the cardiovascular effect of different garlic preparations in diseased, diabetic, and atherosclerotic models. 

## 4. Materials and Methods

### 4.1. Animals

Male Sprague Dawley (SD) rats with an average weight of 575 ± 45 g (*n* = 9, in each groups) were used. Animals were nutrified with standard rodent chow pellets (R/M-Z+H, ssniff Spezialdiäten GmbH, Soest, Germany) ad libitum with free access to water and kept at an ambient temperature of 25 ± 2 °C, with a relative humidity of 55 ± 5%, and a 12-h light-dark cycle. All animals were treated according to the “Principles of Laboratory Animal Care” formulated by the National Society for Medical Research, and the “Guide for the Care and Use of Laboratory Animals” prepared by the National Academy of Sciences and published by the National Institutes of Health (NIH Publication number 86–23, revised in 1996). Breeding and handling of the animals was approved by the Institutional Animal Care and Use Committee of the University of Debrecen, Debrecen, Hungary (May 2012; 12 March 2012).

### 4.2. Treatment Protocol

Rats were randomly segregated into three treatment groups as described: GROUP I: control rats, gavage-treated with the mucin-water vehicle (2% hydroxyethylcellulose solution). GROUP II: RG-treated rats, gavaged with mucin-water, supplemented with 300 mg/kg/day dose of RG. GROUP III: ABG-treated rats, gavaged with mucin-water, supplemented with 300 mg/kg/day dose of ABG. The dose of 300 mg/kg is approximately equal with 15–20 cloves of garlic. Hence, it is rather comparable to 2–3 commercially available garlic capsules or tablets.

All animals were treated every day for a period of 4 weeks. Body mass was measured at the end of treatments. 

### 4.3. Preparation of Aged Garlic

Separated and peeled RG cloves were vacuum sealed in heat-resistant plastic bags. After 3 weeks incubation at 75 °C, conversion was completed and the ABG cloves were used for the treatment and GC-MS analyses.

### 4.4. GC-MS Analyses

Ground raw garlic and aged black garlic cloves were placed into head space vials and were thermostated at 50 °C for 1 h. Solid phase micro-extraction was carried out by using a Supelco fiber assembly with 85 µm polyacrylate-fused silica fiber.

Chromatograms of RG and ABG were taken by a Hewlett-Packard 5890 Series II gas chromatograph-5971A mass spectrometer. Samples were injected into HP-5 stationary phase containing capillary column (25 m × 0.25 mm × 0.25 μm) where the eluent gas was 40 °C helium with 1 mL/min constant flow. Temperatures during analyses were the following: 55 °C for 2 min followed by the scan period to 200 °C with 20 °C/min heating. The overall time of one analysis was 27 min. The temperature of the injector was 200 °C and contained an unpacked liner. Transfer line temperature was 280 °C. Ionization was reached at 70 eV and 10–500 AMU weighed particles were analyzed. Operation of the GC-MS setup, data collecting, and evaluation process was carried out with Hewlett-Packard GC-MS Chemstation rev.3 software (Hewlett-Packard Company, Wilmington, DE, USA. Regarding the mass spectra, components were identified by databases of Nist98 and Wiley. 

### 4.5. Isolated Working Heart Preparation and Cardiac Function Assessments

Following a 4-week treatment period, 24 h after the last treatment, rats were anesthetized with an intraperitoneal pentobarbital sodium injection (60 mg/kg), with heparin as an anticoagulant (1000 U/kg). Following the induction of deep anesthesia, chest cavities were opened, hearts were excised and placed in ice-cold modified Krebs-Henseleit bicarbonate (KHB) buffer (containing 118 mM NaCl, 5.8 mM KCl, 1.8 mM CaCl_2_, 25 mM NaHCO_3_, 0.36 mM KH_2_PO_4_, 1.2 mM MgSO_4_, and 5.0 mM Glucose) to prevent damage of cardiac tissue. Subsequent to excision, aortas were cannulated, and each heart was perfused with modified KHB buffer at a filling pressure of 100 cm of water, using the non-working (Langendorff) mode of the isolated working heart apparatus for 5 min to flush blood out from the hearts. During the washout period, pulmonary veins were cannulated and heart functions were assessed in working mode at a filling pressure of 17 cm of water with KHB buffer. A total of 10 min of working mode activity was sustained to stabilize the cardiac activity. Upon conclusion of 10 min of working mode perfusion, baseline cardiac parameters were registered, including heart rate (HR), aortic flow (AF), and coronary flow (CF); cardiac output (CO) and stroke volume (SV) were calculated. Next, 30 min of ischemia was induced by closing off atrial inflow and aortic outflow. Upon completion of the ischemic period, reperfusion was initiated by opening the aortic cannula. The first 10 min of reperfusions were conducted in the non-working mode to prevent the development of fatal ventricular arrhythmias. The heart was defibrillated with a square wave impulse if ventricular fibrillation was observed at the onset of the reperfusion. Following the first 10 min of Langendorff reperfusion, hearts were switched to working mode for an additional 110 min. Cardiac parameters were recorded at 30, 60 and 120 min of the reperfusion period to monitor the postischemic recovery of the myocardium. A continuous pressure signal was recorded during the whole experiment with the help of a pressure transducer (ADInstruments, PowerLab, Castle Hill, Australia). HR and AOdP/dt were calculated from the continuously recorded pressure signal. AF was measured by a calibrated flow meter, while CF was assessed by time-collecting the coronary effluent. Cardiac output (CO) was calculated as the sum of AF and CF, while stroke volume (SV) was the ratio of CO and HR. Stroke volume alteration was calculated as a ratio of SV and the baseline of SV [8].

### 4.6. Infarct Size Measurements

To monitor the degree of infarction, triphenyl tetrazolium chloride (TTC) (Sigma-Aldrich, Inc., St. Louis, MO, USA) staining was carried out. Hearts were perfused with 35 mL of 1% TTC solution via the aortic cannula at the end of the reperfusion. Following 10 min, hearts were stored at −20 °C for 24 h, to allow each heart to solidify. A total of 2–3 mm thick sections were made from the stained frozen hearts. Sections were subsequently scanned on an Epson J232D flat-bed scanner, blotted dry and weighed. The infarcted area (unstained tissue remained white) and the risk area (entire scanned section) were measured using planimetry software (Image J, National Institutes of Health, Bethesda, MD, USA). Estimates of infarcted zone magnitude were subsequently obtained by multiplying infarcted areas by the weight of each section. The resulting numbers represented the weight of the risk zone and the infarcted zone. Infarct size was expressed as a ratio of the weight of infarcted tissue and the weight of risk zone (whole heart). The entire area of each section was considered to be an infarcted risk zone, while the numerical extent of each infarcted area was planimetrically calculated and multiplied by the weight of the section. Outcomes were expressed as the ratio of the total infarcted tissue volume to volume of at-risk tissue.

### 4.7. Blood Enzymes

Following 4 weeks of treatment with different garlic preparations or mucin-water vehicle prior to sacrifice, peripheral blood was collected from a left external jugular vein of each animal. Analyses of selected serum analytes was conducted using the Cobas 8000 modular analyzer series (Roche Diagnostics GmbH, Mannheim, Germany). The samples were assayed for content of alanine aminotransferase (ALT), alkaline phosphatase (ALP), aspartate aminotransferase (ASTL) total cholesterol (CHO), low-density lipoprotein cholesterol (LDL), triglycerides (TRIG), high-density lipoprotein cholesterol (HDL), lactate dehydrogenase (LDH), C-reactive protein (CRP), and creatine-kinase-myoglobin (CK-MB). Testing was conducted in the Department of Laboratory Medicine, University of Debrecen, Hungary.

### 4.8. Western Blot Analyses

Approximately 300 mg of heart tissue was lysed in 1 mL isolating buffer (25 mM Tris-HCl, 25 mM NaCl, 1 mM orthovanadate, 10 mM NaF, 10 mM pyrophosphate, 10 mM okadaic acid, 0.5 mM EDTA, 1 mM PMSF, and 1× protease inhibitor cocktail) using a polytron homogenizer. Homogenates were centrifuged at 2000 rpm at 4 °C for 10 min. The supernatant was transferred to a new tube and further centrifuged at 10,000 rpm at 4 °C for 20 min; the resultant supernatant was used as a cytosolic extract. The protein concentration was determined by a BCA Protein Assay Kit (Thermo Scientific, Rockford, IL, USA) using bovine serum albumin (BSA) as the standard. Samples were mixed with Laemmli buffer and boiled for 10 min. A total of 100 μg of protein in each sample was loaded and separated on 12% SDS–PAGE gels (Sigma Aldrich, Schnelldorf, Germany) and then transferred to polyvinylidene difluoride (PVDF) membranes (Bio-Rad Laboratories, Hercules, CA, USA). Following blocking the membranes with 5% of nonfat dry milk powder dissolved in tris-buffered saline buffer with 0.1% Tween 20 (TBST) for 1 h, membranes were incubated with primary antibody solution at 4 °C overnight (HO–1 1/500, Abcam, Cambridge, UK; iNOS, Cell Signaling Technology, Boston, MA, USA). The membranes were washed with TBST 3 times and incubated with horseradish peroxidase (HRP)-conjugated secondary antibody solution (1/2000, Cell Signaling Technology, Boston, MA, USA) for 1 h at room temperature. Subsequent to washing, the membranes were developed using Luminate Forte Western HRP substrate (Millipore, Billerica, MA, USA). Chemiluminescence was detected as well as band intensities were measured by ChemiDoc^TM^ Touch Imaging System and Image Lab software (Bio-Rad Inc., Hercules, CA, USA) [8] and normalized against total protein.

### 4.9. Statistical Analyses

All data are presented as the average magnitudes of each outcome in a group ± standard error of the mean (SEM). Statistical analysis was performed using one-way analysis of variance (ANOVA), followed by Kruskal–Wallis or Dunnett’s multiple comparison tests with GraphPad Prism software for Windows (GraphPad Software Inc., La Jolla, CA, USA). Probability values (*p*) less than 0.05 were considered statistically significant.

## Figures and Tables

**Table 1:** Main compounds of raw and black garlic in the gas phase.

Compounds	Retention Time (min)	Raw Garlic	Aged Black Garlic	Structures
2-acetyl-1-pyrroline	7.6	−	+	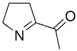
diallyl disulfide	8.0	+	+	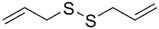
diallyl trisulfide	9.1	+	+	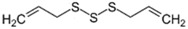
dipropil trisulfide	11.6	+	+	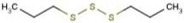
allicin	11.7	+	−	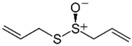

“+” -detectable; “−” non-detectable or just in a very low concentration.

**Table 2:** Blood parameters measured from vehicle-, raw- and aged black-treated animals.

Blood Parameters	Control *n* = 8	Raw Garlic *n* = 10	Aged Black Garlic *n* = 9
ALT (U/L)	45.0 ± 3.3	47.1 ± 1.9	44.1 ± 1.4
ALP (U/L)	91.3 ± 9.2	90.2 ± 1.3	84.8 ± 4.4
ASTL (U/L)	89.1 ± 9.1	89.6 ± 3.8	87.9 ± 4.3
CHO (mmol/L)	1.30 ± 0.12	1.41 ± 0.07	1.58 ± 0.05 *
LDL (mmol/L)	0.224 ± 0.020	0.294 ± 0.019 *	0.350 ± 0.021 *
TRIG (mmol/L)	1.35 ± 0.23	1.07 ± 0.12	1.32 ± 0.10
HDL (mmol/L)	1.09 ± 0.11	1.19 ± 0.04	1.25 ± 0.06
LDH (U/L)	811 ± 173	772 ± 88	801 ± 82
CRP (mg/L)	1.35 ± 0.23	1.07 ± 0.12	1.32 ± 0.10
CK–MB (U/L)	765 ± 154	656 ± 66	746 ± 89

* *p* < 0.05 in comparison with the control group.

## Supplementary Materials

Supplementary materials can be found at http://www.mdpi.com/1422-0067/19/4/1017/s1. Figure S1. Representative Western blots. Upper blot represents protein samples from hearts originating from animals treated with RG, ABG or vehicle were separated, blotted and probed with the HO-1 antibody. Lower blot represents protein samples from hearts originating from animals treated with RG, ABG or vehicle were separated, blotted and probed with the iNOS antibody. Raw garlic (RG); aged black garlic (ABG). BL: baseline samples; I/R: ischemia/reperfused samples.
