# Supplementary figures and images for: Aged (Black) versus Raw Garlic against Ischemia/Reperfusion-Induced Cardiac Complications
Source: Int J Mol Sci. 2018 Mar 28;19(4):1017. doi: 10.3390/ijms19041017 (PMC5979444; doi:10.3390/ijms19041017)

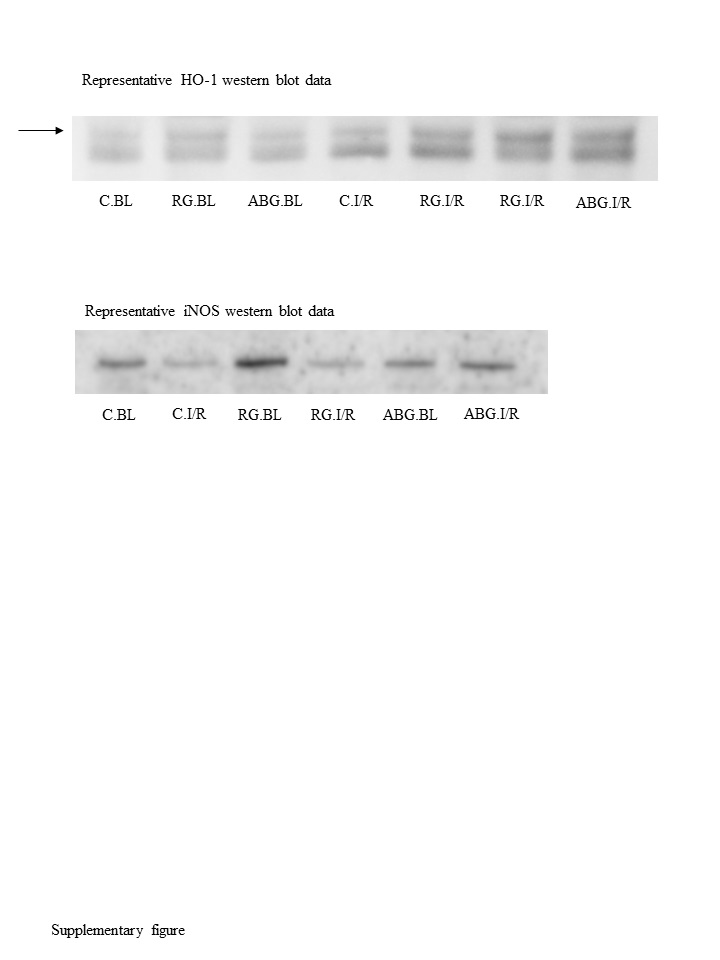

Supplement: Supplementary file 1 [file ijms-19-01017-s001.jpg]
